# Uncovering conserved networks and global conformational changes in G protein-coupled receptor kinases

**DOI:** 10.1016/j.csbj.2024.09.014

**Published:** 2024-09-28

**Authors:** Min Jae Seo, Wookyung Yu

**Affiliations:** a Department of Brain Sciences, DGIST, 333 Techno Jungang-daero, Daegu 42988, Republic of Korea; b Core Protein Resources Center, DGIST, 333 Techno Jungang-daero, Daegu 42988, Republic of Korea

**Keywords:** G protein-coupled receptor kinase, GRK, Conformational change, GPCR, Phosphorylation

## Abstract

G protein-coupled receptor kinases (GRKs) are essential regulators of signaling pathways mediated by G protein-coupled receptors. Recent research suggests that GRK-mediated phosphorylation patterns dictate functional selectivity, leading to biased cellular responses. However, a comprehensive understanding of the structural mechanisms at the single-residue level remains elusive. This study aims to define the general conformational dynamics of GRKs with a particular focus on quantifying the transitions between the closed and open states. Specifically, we examined these transitions, classified based on the ionic lock between the regulatory G protein signaling homology domain and kinase domain. To facilitate a precise structural comparison, we assigned common labels to topologically identical positions across the 47 GRK structures retrieved from the Protein Data Bank. Our analysis identified both general and subfamily-specific dynamic movements within the networks and measured the conformational change scores between the two states. Elucidating these structural dynamics could provide significant insights into the regulatory mechanisms of GRK.

## Highlights

• We defined GRK common Label(GCL) for topological identical residue comparison.
• We compared GRK structures to identify common transitions from closed state(ionic lock) to an open state(lock disrupted).
• We found conserved conformational changes at both residue level and secondary structure level.
• We discovered the underlying mechanism of how GRKs transition into an activated state.

## Graphical Abstract

**Figure:**
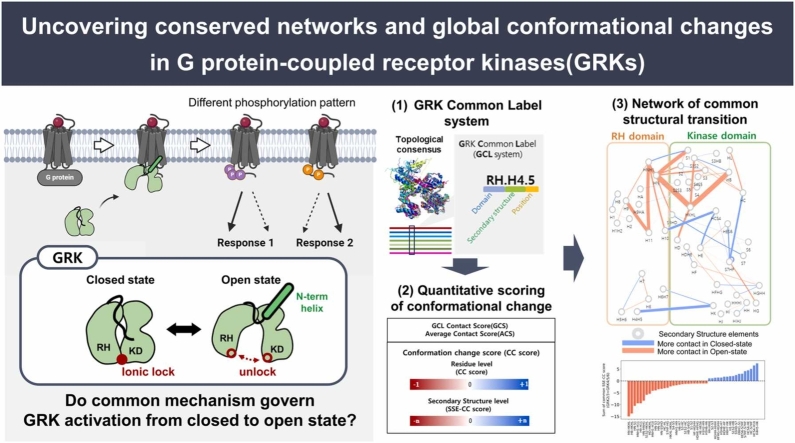

## 1 Introduction

**Fig. 1:**
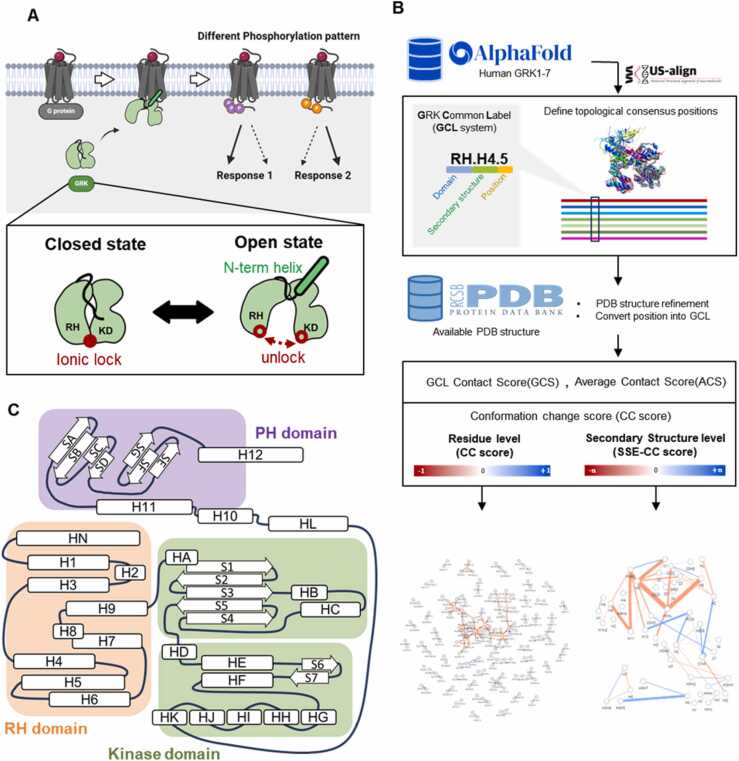
(A) Simple mechanism of G protein-coupled receptor kinase (GRK). The bottom box shows a schematic presentation of GRK in the closed and open states. (B) Simple workflow from common labeling with Alphafold2 structure to contact score networks. (C) Secondary structure element labels according to the GRK Common Label (GCL) system.

G protein-coupled receptor kinases (GRKs) are key proteins that regulate G protein-coupled receptor (GPCR) signaling. During their interactions with GPCRs, GRKs phosphorylate serine/threonine sites in the intracellular region of the receptors, triggering subsequent reactions (Fig. 1 A). Phosphorylation by GRKs is gaining increasing interest owing to its role in the biased signaling of GPCRs. Biased signaling is a phenomenon in which different types of ligands that bind to a receptor activate distinct downstream signaling pathways, leading to various biological outcomes. This process involves two distinct intracellular pathways: one is G protein-mediated signaling, and the other is beta-arrestin-supported signaling [1], [2], [3], [4], [5]. This selective activation can lead to various drug effects, including unexpected side effects. The growing interest in GRKs stems from the fact that their selective activation is driven by the phosphorylation of intracellular loops or the C-termini of GPCRs.

The GRK family comprises seven subtypes, each characterized by distinct features based on subtype classification. These subtypes are further categorized into three subfamilies based on their sequence or functional characteristics. GRK2 and GRK3, as well as GRK4, 5, and 6, share similar sequences within their respective subfamilies (Fig. S1) [6], [7], [8]. The two subfamilies, GRK2/3 and GRK4/5/6, are expressed in peripheral body tissues and the central nervous system (Fig. S2, Supplementary Table 2) [8], [9], [10]. By contrast, GRK1 and GRK7 exhibit relatively independent sequences; their functions are unique, and they are predominantly expressed in the retinal area (Fig. S2). Because GRKs are distributed throughout the body and play a role in numerous physiological processes, they are also associated with various diseases, such as cardiovascular disease, neurodegenerative disorders, and cancer. [11], [12], [13], [14], [15], [16], [17], [18], [19], [20], [21], [22].

GRK subtypes display distinct characteristics reflected in their structural variations. Generally, GRKs share sequences and possess two common domain structures: the regulatory G protein signaling homology (RH) and kinase domains [23], [24]. The RH domain senses GPCR activation and induces conformational changes in the kinase domain, as demonstrated by mutagenesis studies [25]. The kinase domain contains substrate-reactive sites that are crucial for enzymatic activity. Disconnection between the RH and kinase domain causes make the conformational change to an active-like state [23], [26] Additionally, the Pleckstrin Homology (PH) domain facilitates interactions with G protein beta/gamma subunits and is unique to the GRK2/3 subfamily.

Numerous Protein Data Bank (PDB) structures, obtained through either x-ray crystallography or cryo-electron microscopy, have been revealed [26], [27], [28], [29] However, there remains a gap in our understanding regarding the systematic investigation of GRK structures and comparisons across all GRK types.

In this study, we gathered available structural files of GRKs and attempted to compare each PDB file to identify common mechanisms underlying the transition from the closed to open conformation in GRKs. For a precise comparison, we aligned the structures and applied the GRK Common Label (GCL) to each PDB file. Subsequently, we analyzed the common conformational changes across GRK2/3 and GRK4/5/6 subtypes at both the residue and secondary structure levels. Overall, we proposed a unified labeling system for GRKs that facilitates easier comparison of distinct PDB files. Additionally, our study contributes to a deeper understanding of the conformational changes in GRKs from the closed to the open state.

## 2 Materials and methods

### 2.1 GRK common label (GCL)

Full-length GRK structures of human sequences were obtained from the AlphaFold2 database [30]. From GRK1 to GRK7, seven structures (GRK1–7) were superposed via the US-align online web server with multiple structure alignments [31]. We defined topologically identical positions using superposed structures and labeling benchmarks from Flock’s study [32]. Each position received three label sections: domain information, secondary structure element (SSE) information, and position number. Domain information was classified into three categories: the RH, Kinase, and PH domain. SSEs are represented as follows: helices in the RH domain are abbreviated with "H" followed by a number, whereas helices in the KD domain are abbreviated with "H" followed by letters. Beta sheets in the kinase domain are abbreviated with "S" followed by a number, and beta sheets in the PH domain are abbreviated with "S" followed by letters. For example, the RH.H6.3 position refers to the third position of helix six in the RH domain. All GCL labels for human GRK sequences are provided in the attached table file (Supplementary Table 3).

### 2.2 Structure preparation for analysis

A total of 57 PDB files for the structural analysis of GRK were obtained from the RCSB Protein Data Bank.(Supplementary Table 1) We applied a two-step filtering process: first, excluding short fragment GRK sequences, and second, removing PDB structures with a resolution above 4.00 Å. After filtering, 47 PDB structures were obtained. Each chain in the PDB files was then separated into individual PDB files, and the indices were reassigned using the PDB-tools package [33]. This resulted in 61 PDB files for the analysis. Finally, based on the closed-open criteria, GRK2/3 (seven closed states; three open states) and GRK4/5/6 (six closed states; two open states) were selected. The details of the PDB structure codes are provided in Supplementary Table 1.

### 2.3 Contact score calculation

The contact between the residues was defined using two criteria. First, we observed heavy atom contacts, and second, we only considered long-range contacts, where the gap between residues was over 5 positions. If a residue pair aligns with these criteria, a score of 1 is assigned; otherwise, it's assigned 0. Subsequently, we calculated scores at both the residue and SSE levels. At the residue level, the average contact score (ACS) was calculated by summing all the topologically identical positions in the target PDB files and dividing them by the number of PDB files. Similarly, at the SSE level, the contact score of each element was defined as the sum of the residue scores of that element. We then calculated the ACSs of all SSEs across all target PDB files. The conformational change score(CC score) at the residue or secondary structure level was obtained by subtracting the ACS of the open state from that of the closed state. In Fig. 4, Fig. 5, the edge widths represent the sum of the GRK2/3 and GRK4/5/6 CC scores, which exhibit similar tendencies of conformational changes.

### 2.4 Visualization

Data visualization in this study was conducted using Python, employing the matplotlib module for boxplots, scatter plots, histograms, and line plots; the Pyvis module for the network diagram; and Bokeh for the chord diagram [34], [35], [36]. All structural images were generated using the PyMOL software [37].

### 2.5 Sequence and conservation

We gathered sequences for conservation scores from UniProt, applying specific criteria, such as filtering by gene name, vertebrates, and sequence length within a range of ± 50 compared with those of *Homo sapiens* [38]. To ensure impartiality, we exclusively considered sequences common across GRK1 to GRK7, resulting in 37 species sequences for each subtype and 259 sequences, primarily comprising mammalian sequences. The sequences were realigned in a GCL-matched form. The conservation scores depicted in Fig. 5 were generated using Jalview software [39]. Additionally, the sequence identities in Fig. S1 were calculated on the Clustal Omega server at EMBL-EBI using only human sequences [40].

## 3 Results

### 3.1 Identification of conserved ionic lock and GRK state classification using the GCL system

To ensure consistent comparisons across the available GRK structures, we adopted a standardized approach for comparing structurally identical residues, which was first used in a G protein study [32]. We developed a GCL system by applying this approach and assigning a three-part label to each structural position: domain information, SSE, and position number (Fig. 1B). This system allows the comparison of topologically equivalent positions across all seven GRK subtypes. The names of each secondary structural element are shown in Fig. 1 C.

**Fig. 2:**
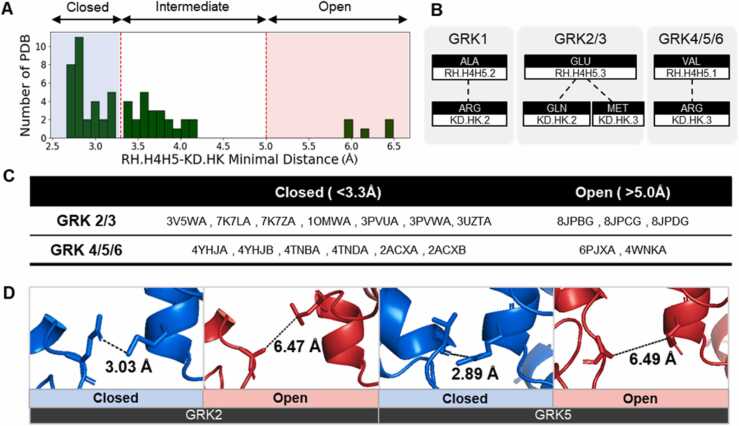
(A) Histogram of the minimal distance between residues in RH.H4H5 and KD.HK. The Y-axis represents the number of Protein Data Bank (PDB) files, and the X-axis represents the distance. These data also include the G protein-coupled receptor kinase (GRK)1 structure. (B) Amino acid information of the ionic lock between RH.H4H5 and KD.HK according to each GRK subfamily. (C) Filtered PDB files according to the selection criteria. The last letter of the PDB file names is the chain identifier. (D) Structures of the closed and open states. From left to right: PDB codes 3PVW, 8JPB, 4TND, and 4WNK.

Based on the GCL system, we analyzed 61 PDB files and treated each chain in a single PDB file as a distinct structure (Fig. S3, Supplementary Table 1). Recent studies have suggested that the GRK ionic-lock residue pairs become distant during physiological reactions [26]. This lock is described as occurring between the bottom loop of the RH domain and the bottom helix of kinase domain, which was designated in the GCL system as RH.H4H5.1 and KD.HK.3. Therefore, we measured the minimal distance between RH.H4H5 and KD.HK in each PDB file to determine the closed-open state. The open state of GRK files can be clearly observed to be over 5.0 Å in Fig. 2A. Conversely, the closed state is defined as a distance under 3.3 Å between RH.H4H5 and KD.HK. We selected 13 structures for the closed state, comprising seven structures for GRK2/3 and six structures for GRK4/5/6. In addition, five structures were chosen for the open state, comprising three structures for GRK2/3 and two structures for GRK4/5/6 (Fig. 2C). Examples are shown in Fig. 2D. The GRK1/7 subfamilies were excluded from the analysis on conformational change due to their specialized expression patterns (Fig. S2) and the absence of open state structures.

The conservation of this ionic lock, or the closest contact between them, varies among the subfamilies. In GRK1, the closest contact is between RH.H4H5.2 (Ala) and KD.HK.2 (Arg). For GRK2/3, RH.H4H5.3 (Glu) is observed to have the closest contact with either KD.HK.2 (Gln) or KD.HK.3 (Met). In GRK4/5/6, the closest contact occurs between RH.H4H5 (Val) and KD.HK.3 (Arg) (Fig. 2B). GRK7 is excluded due to the absence of an available structure.

### 3.2 Quantitatively measurements of conformational change between states

**Fig. 3:**
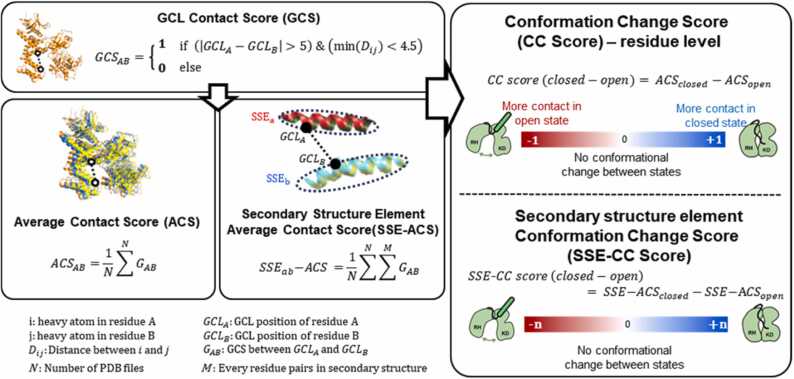
Flow of contact score calculation. G protein-coupled receptor kinase (GRK) Common Label (GCL) Contact Score (upper left box): long-range heavy atom contacts at GCL positions in GRK structures. Average Contact Score (ACS) and Secondary Structure Element (SSE)-ACS (lower left boxes): contact scores of residues and secondary structure elements, respectively. The right box shows the conformational change (CC) score at the residue level and secondary structure level. A score of zero indicates no contact change between the closed and open structures.

To explore the shared movements between the closed and open states, we categorized our targets into four classes: GRK23-closed, GRK23-open, GRK456-closed, and GRK456-open. We calculated the contact of each GCL position represented by a binary value called the GCL Contact Score (Fig. 3). We then determined the Average Contact Score (ACS), which is the average GCL Contact Score in the target structure files. Contacts between SSEs were also calculated, yielding the Secondary Structure Elements Average Contact Score (SSE-ACS). Additionally, we aimed to track the transition from a closed to an open state by quantifying it as the Conformational Change Score (CC score) at both the residue and SSE levels. Detailed concepts and explanations are provided in Fig. 3 and the Methods section, respectively.

The distributions of SSE-ACS for both GRK2/3 and GRK4/5/6 were similar and predominantly concentrated near zero, indicating few contacts between the two SSEs, whereas higher values indicate that the two SSEs were adjacent (Figs. S4A and 4B and E) Additionally, the SSE-CC distribution follows a similar pattern in both GRK2/3 and GRK4/5/6 (Fig. S4C and D). This tendency is also evident in residue-level analyses. ACS at the residue level shows a similar distribution in both GRK2/3 and GRK4/5/6 (Figs. S5A and 5B). However, it is important to note that the total number of contacts is higher in GRK2/3, regardless of the SSE or residue level, owing to the presence of the additional PH domain. Additionally, the distribution of CC-score of both subfamilies is similar (Figs. S4C, 4D, 5C, and 5D). The histograms show a bell-shaped distribution with most data points near zero; however, high values, although less frequent, are crucial as they represent significant conformational changes.

### 3.3 Secondary structure movements are conserved within subfamilies

**Fig. 4:**
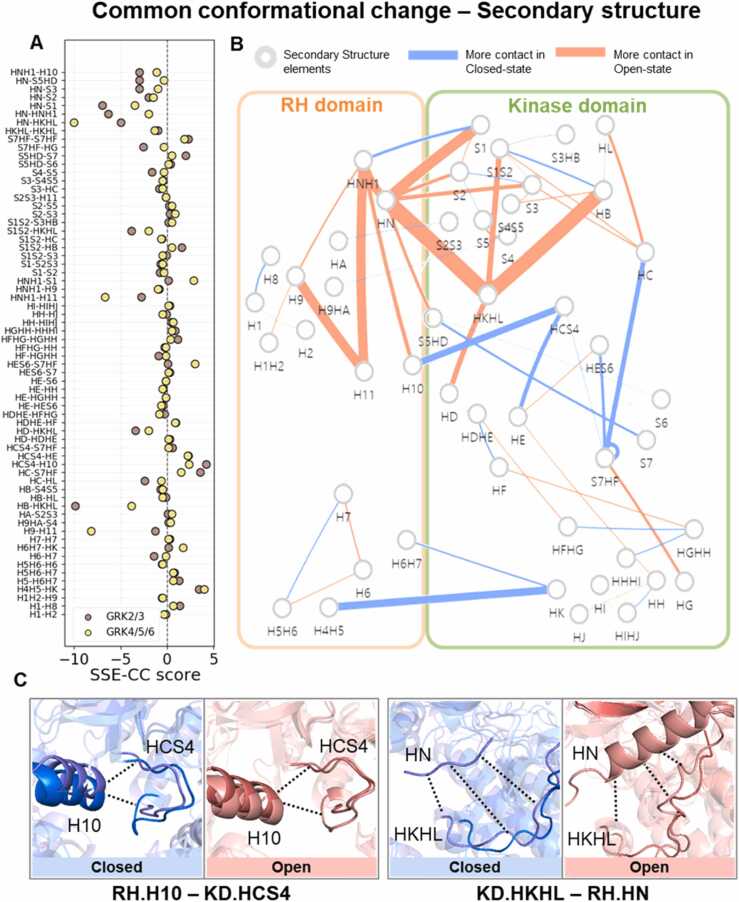
(A) Conformational change of each G protein-coupled receptor kinase (GRK)2/3 and GRK4/5/6 subfamily. The X-axis represents the secondary structure element-conformational change (SSE-CC) score. The Y-axis represents secondary structure pairs showing common conformational movements. (B) Network showing the sum of GRK2/3 and GRK4/5/6 SSE-CC scores. Thicker edges indicate a higher tendency for contact in either the closed or open state (Fig. S7B). Each node represents a secondary structure element. (C) Example of conformational differences between closed and open states. Only common movements are shown in the network. In the left box, RH.H10- KD.HCS4 interactions are more prominent in the closed state. In the right box, KD.HKHL-RH.HN interactions show closer contact in the open state.

Across the various structures in different subfamilies, 301 SSE interaction pairs were observed, of which 105 pairs showed the same tendency, indicating that they exhibited the same positive or negative sign in their contact score patterns during transitions. Approximately 30 % of paired movements were observed (Fig. S7). We focused on the common interaction pairs that exhibited similar tendencies across different subfamilies, highlighting their role in representing the global movement of GRK state transitions (Fig. 4, Appendix A, Appendix A).

To further explain this, we excluded interactions with opposing SSE-CC scores (one positive and one negative) between each subfamily because such discrepancies likely indicate biases rather than representing global movements. At the secondary structure level, most pairs had similar SSE-CC scores, indicating that their levels of conformational changes were comparable. However, certain SSEs exhibited different dynamics, with some interactions showing higher SSE-CC scores than those in other subfamilies, suggesting that these interactions are more dynamic within that particular subfamily (Fig. 4 A). For example, interactions involving KD.S1, KD.S1S2, or KD.S2 generally displayed similar SSE-CC scores for GRK2/3 and GRK4/5/6, indicating comparable conformational dynamics. However, in the case of the RH.HN-KD.HKHL interaction, pairs of SSEs showed negative SSE-CC scores in both subfamilies, with a more negative score observed in GRK4/5/6 than in GRK2/3. This suggests that the distance between HN and HKHL is smaller in the open state for both subfamilies; however, the conformational gap is larger in the GRK4/5/6 subfamily.

Presenting the scores in networks (Fig. 4B and Appendix A, Appendix A), it is evident that interactions between RH.H4H5-KD.HK occur in closed states, as this was a criterion for state classification. Notably, RH.HN and KD.HKHL serve as hubs. The KD.HKHL loop is the longest loop in GRK, spanning from the bottom to the upper region of the kinase domain, almost traversing the side of the kinase domain. The KD.HKHL loop contacts KD.HB, KD.HD, KD.S1S2, and RH.HN. Among these, the KD.HKHL-RH.HN interaction is particularly specific to the open structure, as the RH.HN helix is part of the N-terminal region and does not adopt a helical form in the closed state (Fig. 4 C, right). In the open state, the N-terminal loops form a helix and contact KD.HKHL and KD.S1.

Another interesting observation is the increased contact between KD.HKHL and KD.HD in the open state. Simultaneously, the elements comprising the middle part of the kinase domain, such as KD.HC, KD.HCS4, KD.HE, KD.HES6, and RH.H10, showed increased contact in the closed state (Fig. 4 A). In other words, the contacts between the middle parts of the kinase domain were disrupted in the open state, suggesting that the lobes of the kinase domain are distanced. By contrast, the KD.HD helix located around the hinge region of the kinase domain maintains contact in the open state.

An interesting finding within the RH domain is the interaction of the RH.HNH1 region with RH.H11, RH.H10, and KD.S5HD and its subsequent connection with RH.H9 in the open state. This contact network extends to the upper region of the kinase domain, indicating that the structural transition mechanism involves communication with the upper regions of both the RH and kinase domains.

### 3.4 Residue-level movement is conserved via evolutionarily conserved residues

**Fig. 5:**
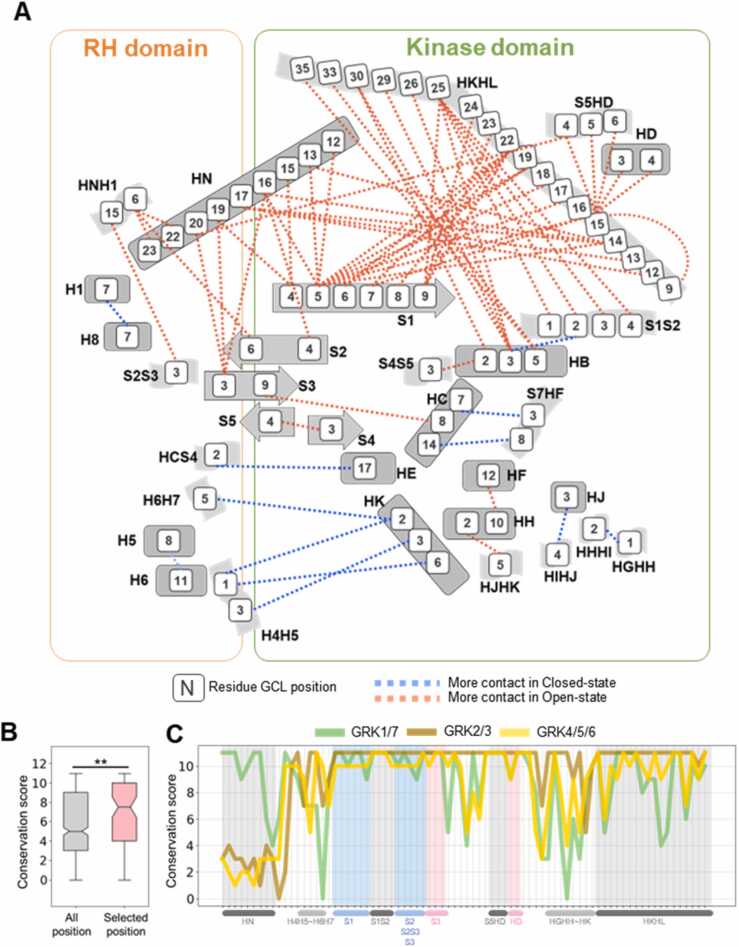
(A) Conformational changes at the residue level. Each square represents a residue at a specific G protein-coupled receptor kinase (GRK) Common Label (GCL) position in the GRK structure. Only residues showing common conformational movements are presented. (B) Conservation scores of all positions and those shown in (A), calculated by Jalview software. Statistical analysis was performed using the Mann–Whitney U test (*: p-value < 0.1, **:p-value < 0.01, ***:p-value<0.001). (C) Conservation scores of each of the 78 GCL positions in (A), categorized by GRK subfamily. The X-axis labels for GCL positions are provided in the attached files.

Conserved global conformational movements were observed at the residue level. The nodes and edges represent the sum of the CC scores in GRK2/3 and GRK4/5/6 exceeding one, while ignoring other subtle conformational changes (Fig. 5 A). The strengths of the contacts are illustrated in Fig. S8.

We compared the major 78 residues involved in the closed-open transition to the overall positions and found that these 78 residues were significantly more conserved than the overall conservation (Fig. 5B). The conservation measurements primarily encompassed the sequences of 37 species related to humans. The conservation at each GCL level is depicted in Fig. 4 C. Notably, the N-terminal regions showed lower conservation than that of the other regions. Interestingly, although loop regions are generally known to be less conserved than helices or sheets, in this case, the loop regions were also highly conserved when involved in major structural transitions (Fig. 4 C and Appendix A, Appendix A).

These trends extended to each subfamily. Within each subfamily, 78 positions showed notably higher conservation than the overall positions, as depicted in Figs. S9A-C. Moreover, at each GCL position (Fig. 4 C and Appendix A, Appendix A), the primary patterns within each subfamily were closely aligned. From these 78 positions, it was evident that the N-terminal regions generally exhibited lower conservation, except for the N-terminal region of GRK1/7, which showed relatively higher conservation. Furthermore, the lower region of the kinase domain, such as from the KD.HGHH loops to the KD.HK helix, demonstrated fewer conservation patterns. Similarly, the lower region of the RH domain, exemplified by regions, such as RH.H4H5 and RH.H6H7, exhibited lower conservation. Conservation of loops within the GRK structure was also consistently observed across each subtype.

Residue-level analysis revealed several intriguing points. Certain residues appear to be crucial for forming contacts with others. For instance, KD.HKHL.25 demonstrates extensive interactions with KD.S1, KD.HB, and KD.S1S2. The KD.HKHL.25 is highly conserved as a phenylalanine and interacts with the conserved residues of KD.S1, KD.HB, and KD.S1S2. Specifically, KD.HKHL.25 contacts KD.S1S2.1–4 with conserved glycine or phenylalanine residues. Notably, the interaction of KD.HKHL.25 with HB.3 (Arg) and S1.9 (Arg, Lys) showed that phenylalanine interacts with positively charged amino acids. Additionally, the numerous contacts between KD.S1.5, KD.KHKL.14, and other residues aligned well with the mutagenesis results, suggesting their role in regulating kinase activity [41].

Intriguingly, KD.HB.3 and KD.S1.9 establish numerous contacts in the KD.HKHL loops, suggesting their roles in stabilizing these loops. Another residue in S1, S1.5 (Arg), also showed positively charged conservation and contacted multiple positions in KD.HKHL. This pattern suggests that positively charged positions within kinase domain play a significant role in establishing contacts in the open-state structure. To further explain this phenomenon, the relative solvent-accessible surface areas of these regions were calculated. We found that the residues were more exposed when the structures were in their closed states and hidden when they were in their open states because of the HKHL loops (Fig. S9E).

Regarding the ionic lock between two domains, residues RH.H4H5.1 and RH.H4H5.3, as well as KD.HK.2, KD.HK.3, and KD.HK.6, are involved. This interaction extends beyond RH.H4H5 and KD.HK, encompassing RH.H6H7, KD.HCS4, and KD.HE in a closed state. Additionally, in the open state, KD.HC.8 (mostly conserved as Glu) interacts with KD.S3.9 (conserved as Leu) situated in the upper region of the kinase domain. Conversely, in closed states, KD.HC.7 (mostly conserved as Asn) and KD.HC.14 (mostly Leu or Lys) interact with KD.S7HF.3 and KD.S7HF.8, which are located in the lower region of the kinase domain.

Overall, the conservation pathway of the structural transitions between the closed and open states of the GRKs was elucidated by calculating the CC scores at the residue and SSE levels.

### 3.5 PH domain still plays a crucial role in structure changes in GRK2/3 subtypes

As previously discussed, the PH domain is unique to GRK2/3 and plays a crucial role in interfacing with the G protein beta and gamma subunits. Because it is specific to these subtypes, we conducted a separate analysis of this domain. At the residue level, the CC score distribution of the intra-PH domain interactions mirrored the trend observed in other domains (Fig. S11A). Notably, these patterns predominantly lean towards positive values, suggesting a denser arrangement of the PH domains in the closed state. Furthermore, when exploring the PH domain interactions with other domains, we observed a reduction in scale; however, the characteristic bell-shaped distribution of the number of contact pairs persisted (Fig. S11B). This highlights the importance of shifts in the PH domain interactions between the closed and open states, which still play a crucial role in shaping GRK's structural changes over time.

At the secondary structure level (Fig. S11C), the H12 helix in the PH domain emerges as a central hub of closed-state PH domain contacts, engaging with various other elements. In particular, its connection to the H1 helix in the RH domain suggests that it plays a role in maintaining the structure in the closed state. Another notable interaction involves the PH.H11SA loop, which acts as a crucial bridge between the RH and PH domains. In the closed state, this loop anchors the PH domain to the interface with the beta-sheet PH.SB. However, during the transition to the open state, PH.SB disengages from the PH.H11SA region and forms robust interactions with other beta-sheets within the domain, indicating dynamic structural rearrangements.

## 4 Discussion

We aimed to establish a universal standard for the conformational changes in GRKs that can be applied across all GRK subtypes, rather than being limited to one or two specific structures. Although numerous studies have elucidated the structural characteristics of GRKs using experimental or computational methods, there has been no systematic effort to merge the information and characteristics of GRKs from available structures [24], [26], [27], [28], [29]. In this study, we present an advancement in our understanding of the structural movements of GRKs from closed to open states.

Our findings show that specific residues and SSEs are more engaged in closed states, whereas others are more involved in open states. The states were classified based on the distance between RH.H4H5 and the KD.HK helix, involving a previously known contact called an ionic lock [26]. We propose that common contact changes during structural transitions occur through conserved residues. Interestingly, although the conserved residues varied, certain loop regions exhibited high conservation and served as hub residues in the networks in the open state. Global structural transitions were also observed in the secondary structures. At the secondary structure level, certain elements, such as the RH.HN helix, KD.HKHL loop, and KD.HB helix, act as transition hubs.

Regarding the PH domain, we focused on studying the conformational changes within the PH domain itself, rather than comparing it to other domains because it is unique to GRK2/3. Shifts between the closed and open states in the PH domain affect the overall structure of GRK, with key elements, such as the PH.H12 helix and PH.H11SA loop, playing essential roles in maintaining the closed state and supporting transitions.

Although we have provided insights into the universal mechanisms underlying the transition of GRKs from the closed to open state, this study has some limitations. Specifically, we analyzed only structures that met strict criteria to eliminate ambiguity. This allowed us to focus exclusively on clear closed and open states while excluding any intermediate structures. Additionally, the limited availability of open-state structures may have resulted in more condensed networks for open-state contacts, although this is not direct evidence of greater consensus in the open states. Furthermore, the process of separating chains from a single PDB file may introduce bias, although filtering to include only common contacts may help minimize this bias.

Several aspects remain to be investigated in the future. We explored common conformational rearrangement mechanisms; however, the lack of a GPCR complex analysis presents a challenge in directly linking our findings to receptor interactions. A hypothesis that emerges, although based on limited evidence, is that activation triggering and regulation may be influenced by variations in N-terminal sequences. For instance, we noted that GRK1/7 showed greater sequence conservation relative to other subfamilies, suggesting a more limited receptor target range. By contrast, GRK2/3 and GRK4/5/6 exhibit lower sequence conservation, indicating a broader receptor spectrum. Future studies should include sequence analyses of the N-terminus and its co-evolution with GPCRs to shed light on the direct mechanisms underlying GPCR recognition. Additionally, uncommon interactions may help analyze the interactions of subfamilies with receptors.

Our findings on the conserved mechanisms underlying GRK function provide a solid foundation for understanding the structural transitions of GRKs from the closed state to the open state. This knowledge offers valuable insights into how GRK movements occur and opens the door for further investigation into target-specific phosphorylation mechanisms. By elucidating the structural dynamics of GRK activation, our study lays the groundwork for deeper exploration of how GRKs regulate receptor phosphorylation and signaling pathways.

## Author statement

All authors have read and approve the submission of this manuscript to the journal of computational and structural biotechnology. All authors declare no conflict of interest.

## CRediT authorship contribution statement

**Wookyung Yu:** Writing – review & editing, Supervision, Project administration, Funding acquisition, Conceptualization. **Min Jae Seo:** Writing – review & editing, Writing – original draft, Validation, Investigation, Formal analysis, Data curation, Conceptualization.

## Declaration of Generative AI and AI-assisted technologies in the writing process

During the preparation of this work the authors used chatGPT in order to edit English as non-native English speaker. After using this service, the authors reviewed and edited the content as needed and take full responsibility for the content of the publication.

## Declaration of Competing Interest

The authors declare no conflicts of interest..

## Footnotes

Appendix A Supplementary data associated with this article can be found in the online version at doi:10.1016/j.csbj.2024.09.014.
