# Supplementary material for: Uncovering conserved networks and global conformational changes in G protein-coupled receptor kinases
Source: Comput Struct Biotechnol J. 2024 Sep 28;23:3445–53. doi: 10.1016/j.csbj.2024.09.014 (PMC11472376; doi:10.1016/j.csbj.2024.09.014)
Supplement: Supplementary file 1 — Supplementary material [file mmc1.pdf]

Supplementary Figures

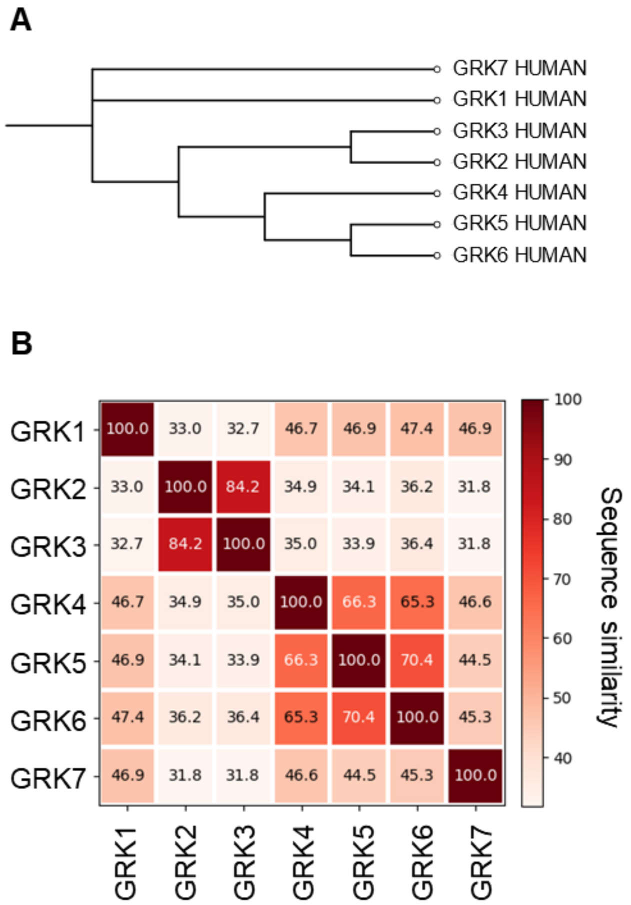

Supplementary figure 1. (A) G protein-coupled receptor kinase (GRK) subtype classification by sequence similarity using Clustal Omega server at EMBL-EBI. (B) Sequence identity matrix of GRK.

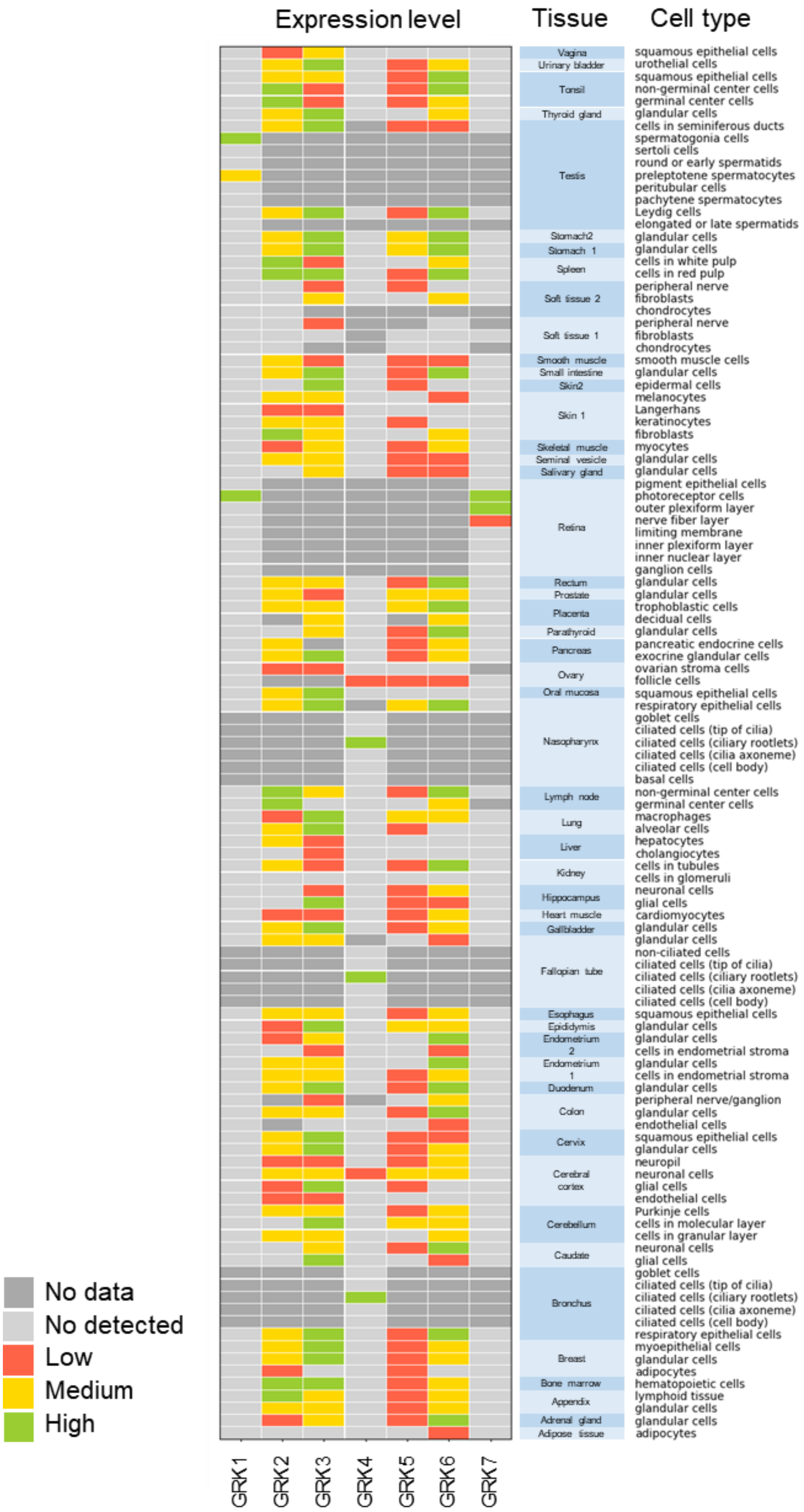

Supplementary figure 2. Tissue expression and cell type expression of GRK. Data were gathered from Human protein atlas.[1,2]

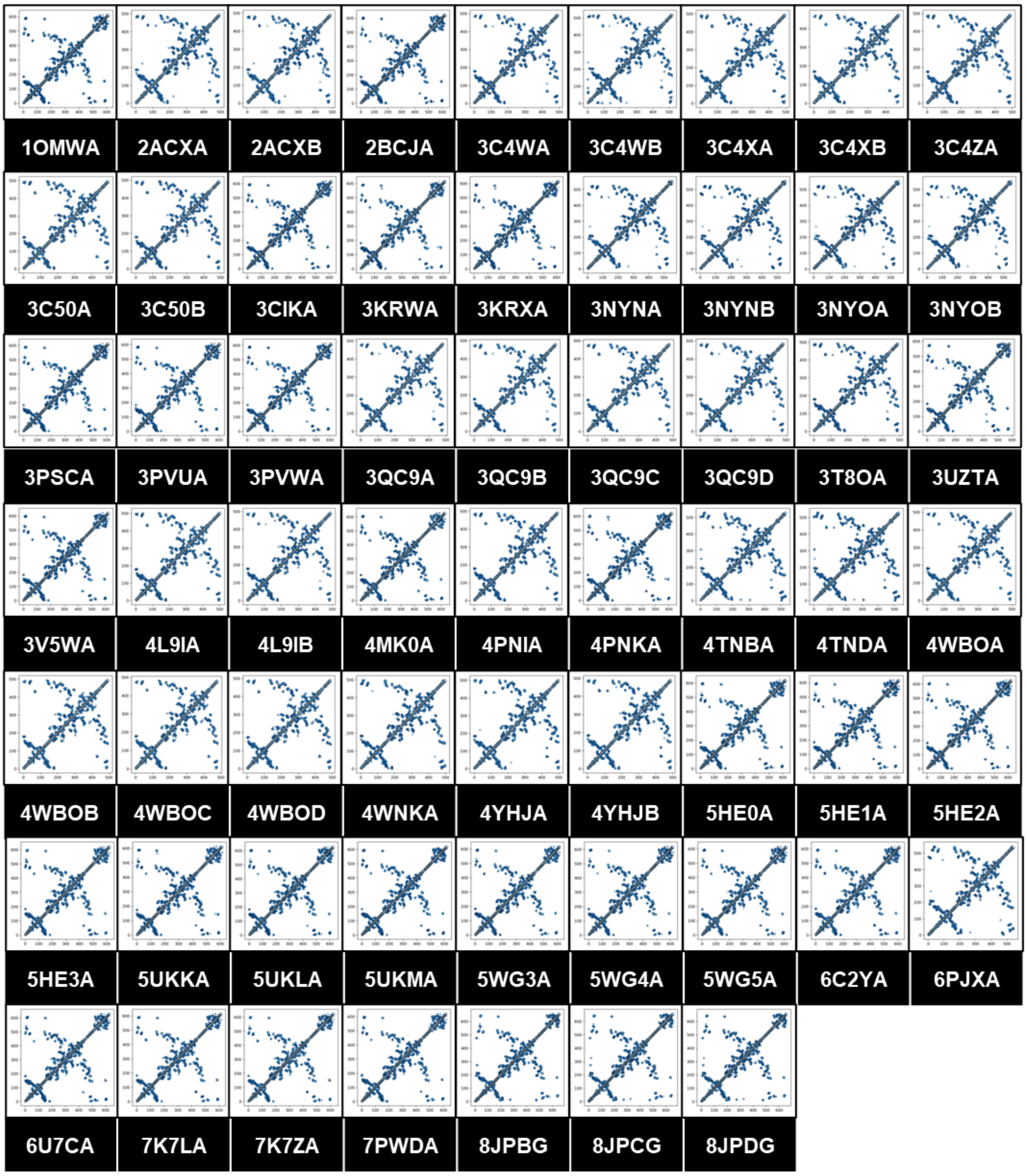

Supplementary figure 3. Contact map of available GRK structures. Each PDB file are separated by chain. The last letter present chain identifier.

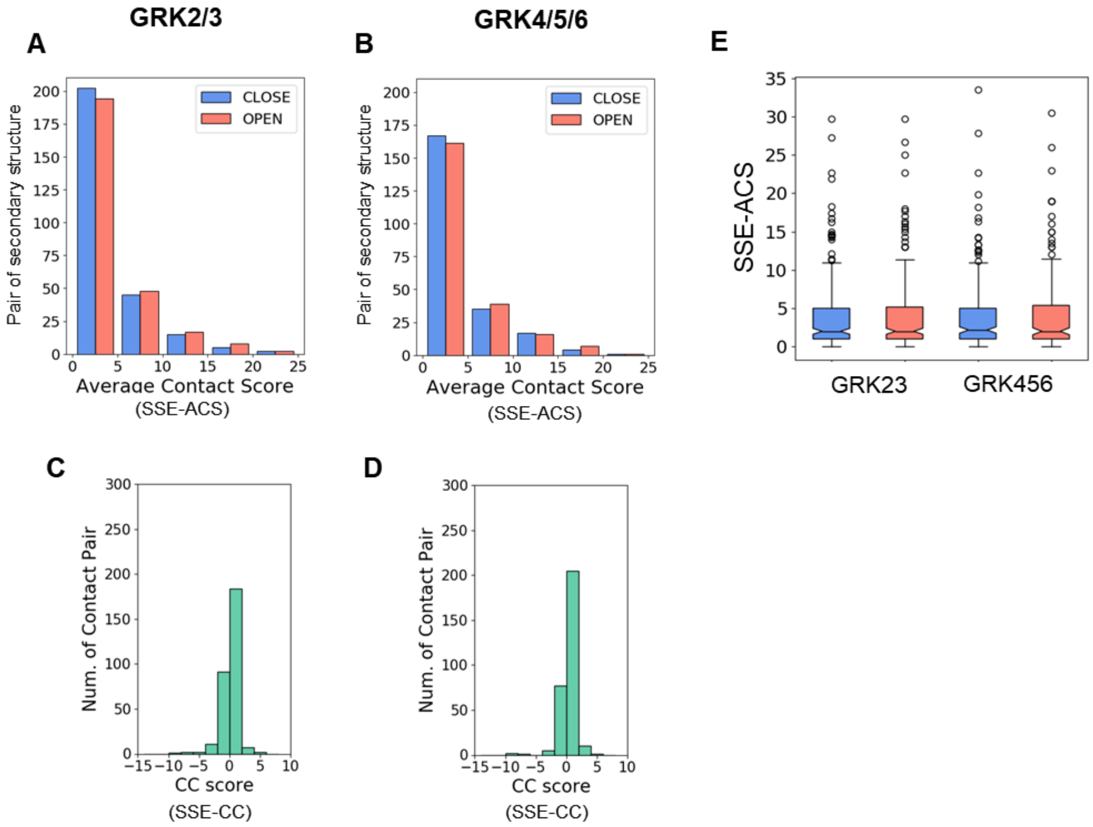

Supplementary figure 4. (A) Number of residue pairs of GRK2/3 closed and open state structure. X-axis means Secondary Structure Element-Average Contact Score(SSE-ACS). (B) Number of residue pairs of GRK4/5/6 closed and open state structure. X-axis means SSE-ACS. (C) Histogram of GRK2/3 conformational change score at secondary structure level. (D) Histogram of GRK4/5/6 Secondary Structure Element-Conformational Change. (SSE-CC). (E) GRK2/3 and GRK4/5/6 showed similar distribution of SSE-ACS. Blue boxes are closed state and red boxes are open state.

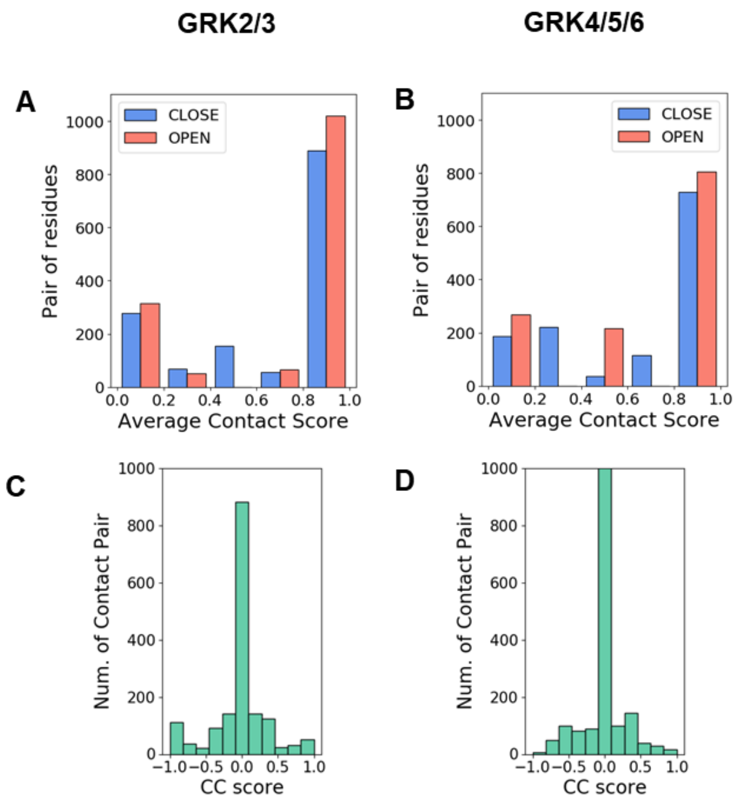

Supplementary figure 5. (A) Number of residue pairs of GRK2/3 closed and open state structure. X-axis means Average Contact Score (ACS) at residue level (B) Number of residue pairs of GRK4/5/6 closed and open state structure. X-axis means ACS at residue level. (C) Histogram of GRK2/3 Conformational Change score (CC score) at residue level. (D) Histogram of GRK4/5/6 CC score at residue level.

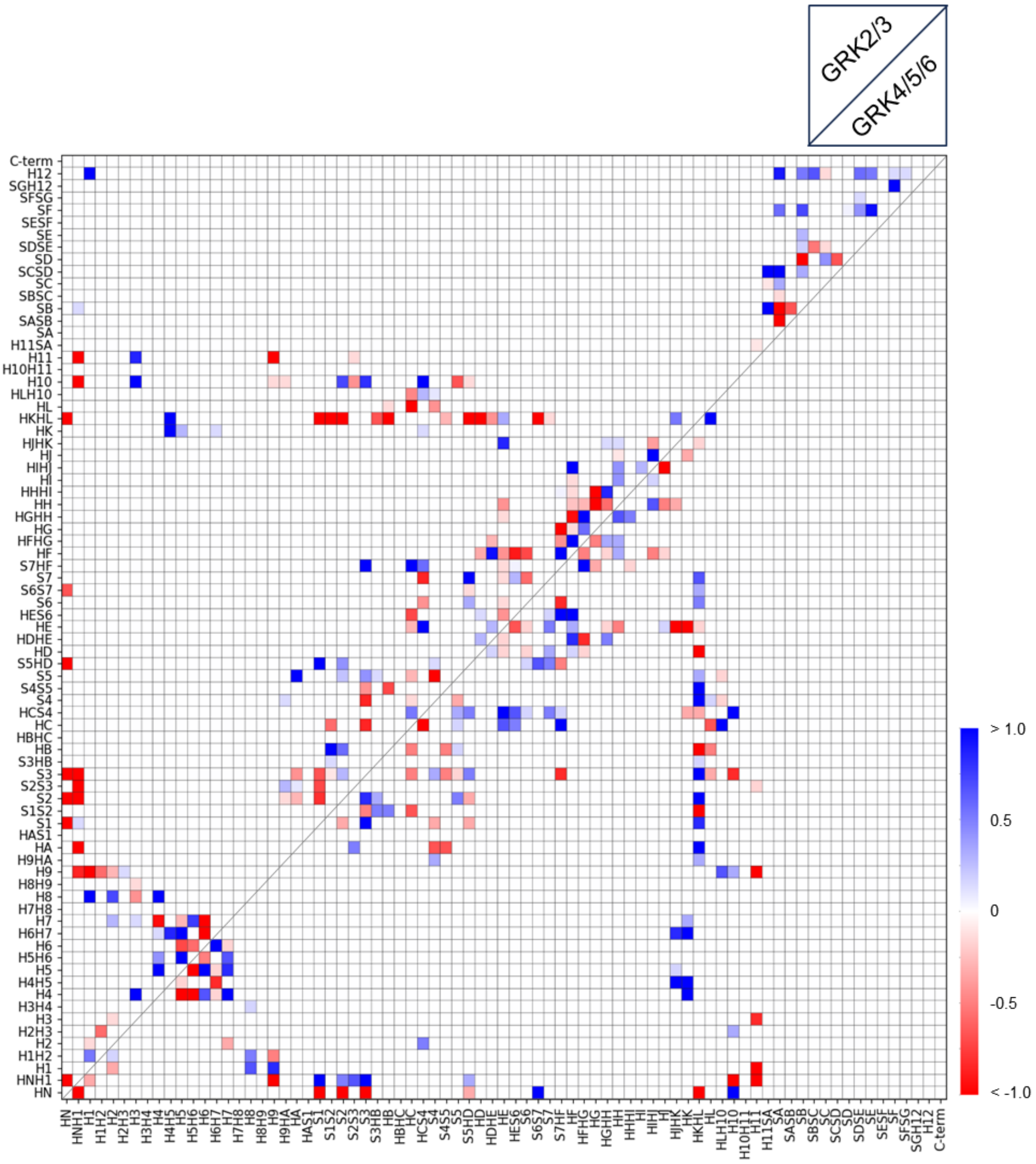

Supplementary figure 6. Secondary Structure Element-Conformational Change score of GRK2/3(Upper triangle) and GRK4/5/6(Lower triangle)

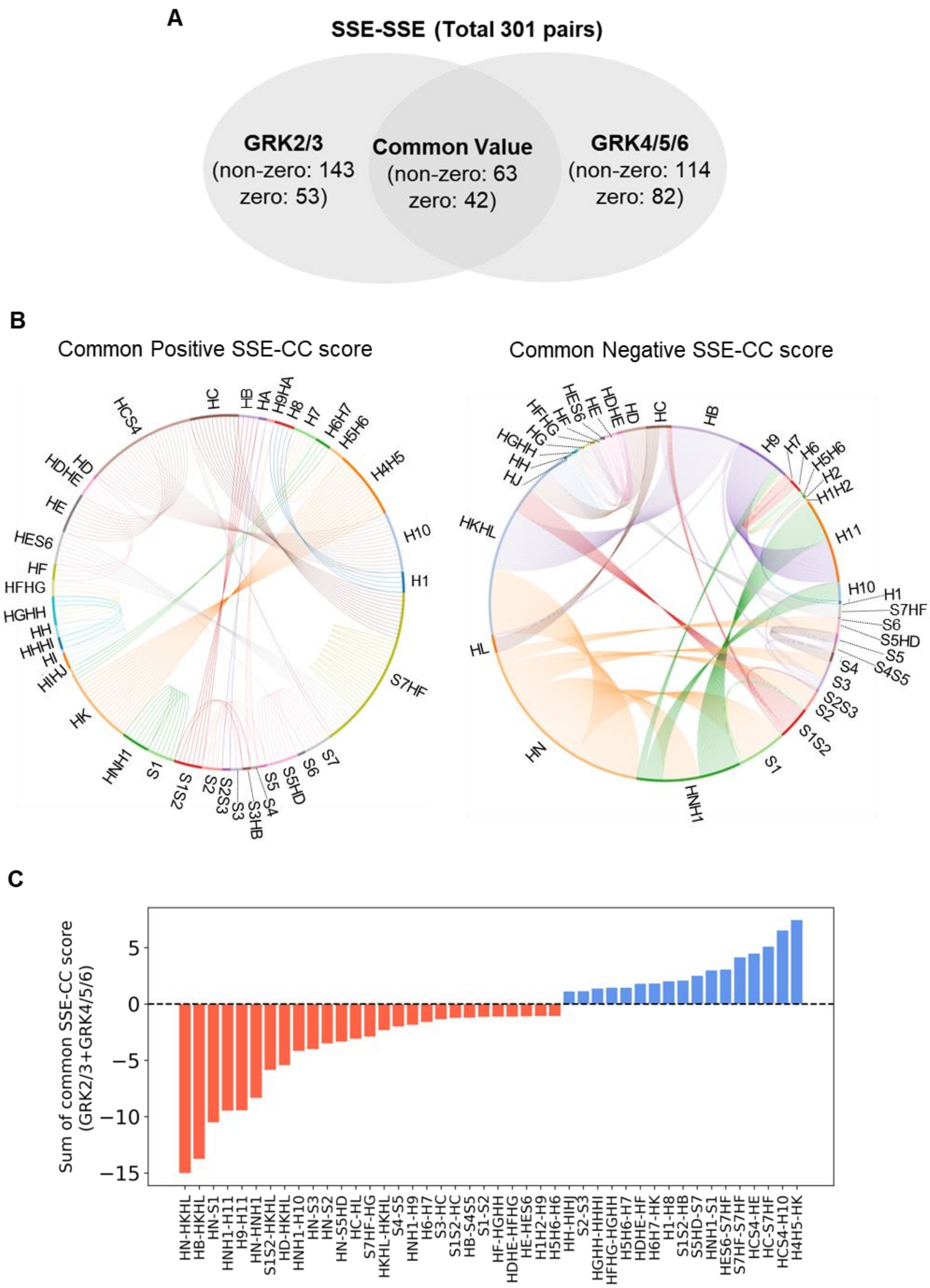

Supplementary figure 7. (A) Chord diagram of common positive-negative Secondary Structure Element-Conformational Change (SSE-CC) score of GRK2/3 and GRK4/5/6. (B) Bar plot of common SSE-CC score in Fig4B.

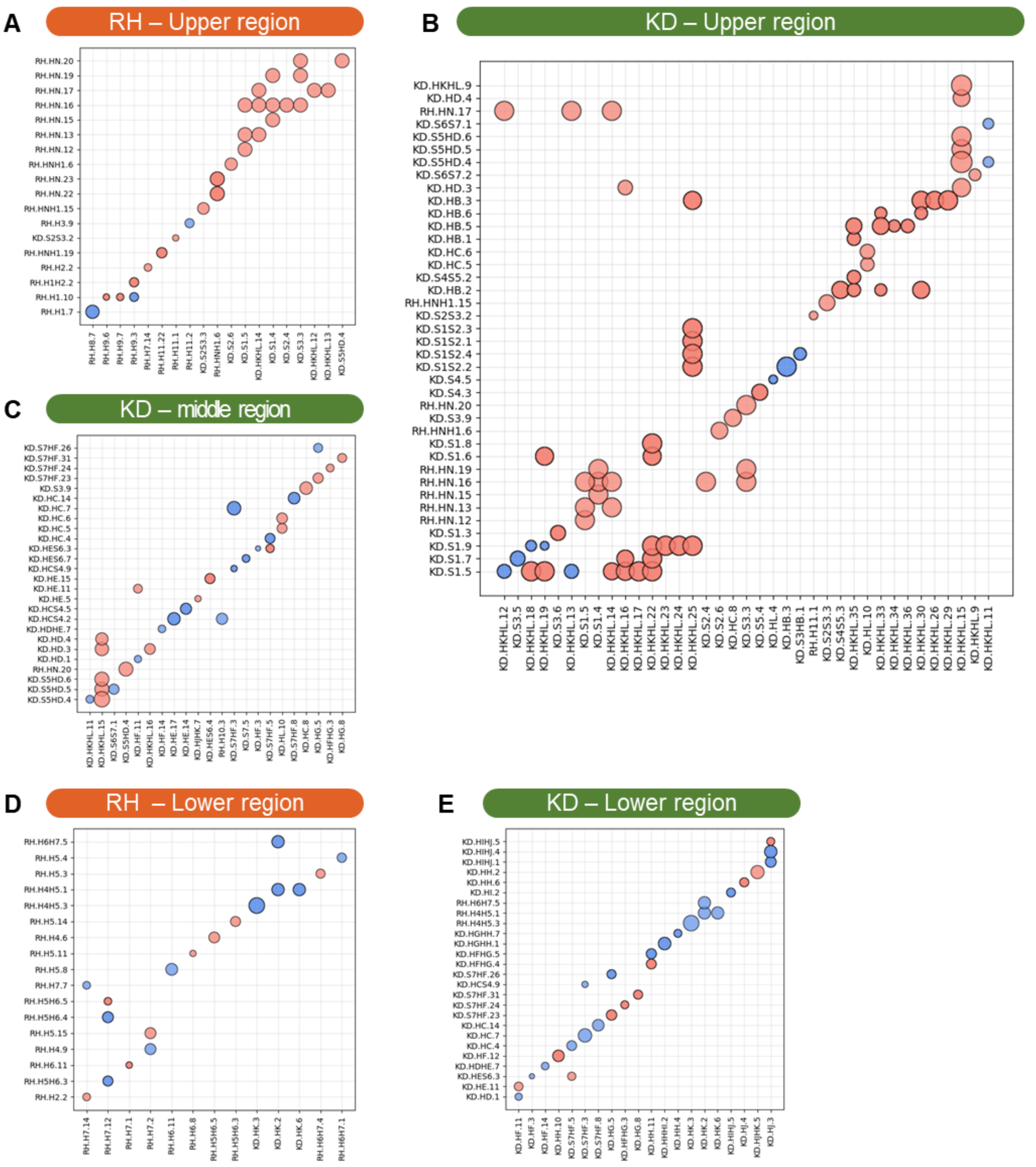

Supplementary figure 8. Scatter plot of Average Contact Score(ACS) between GRK Common Label(GCL) positions. Only common conformational movements across GRK2/3 and GRK4/5/6 are showed. The size of circles present ACS score. (A) Residue pairs involve Upper RH domain region (B) Residue pairs involve Upper Kinase domain region (C) Residue pairs involve Middle Kinase domain region (D) Residue pairs involve Lower RH domain region (E) Residue pairs involve Lower Kinase domain region

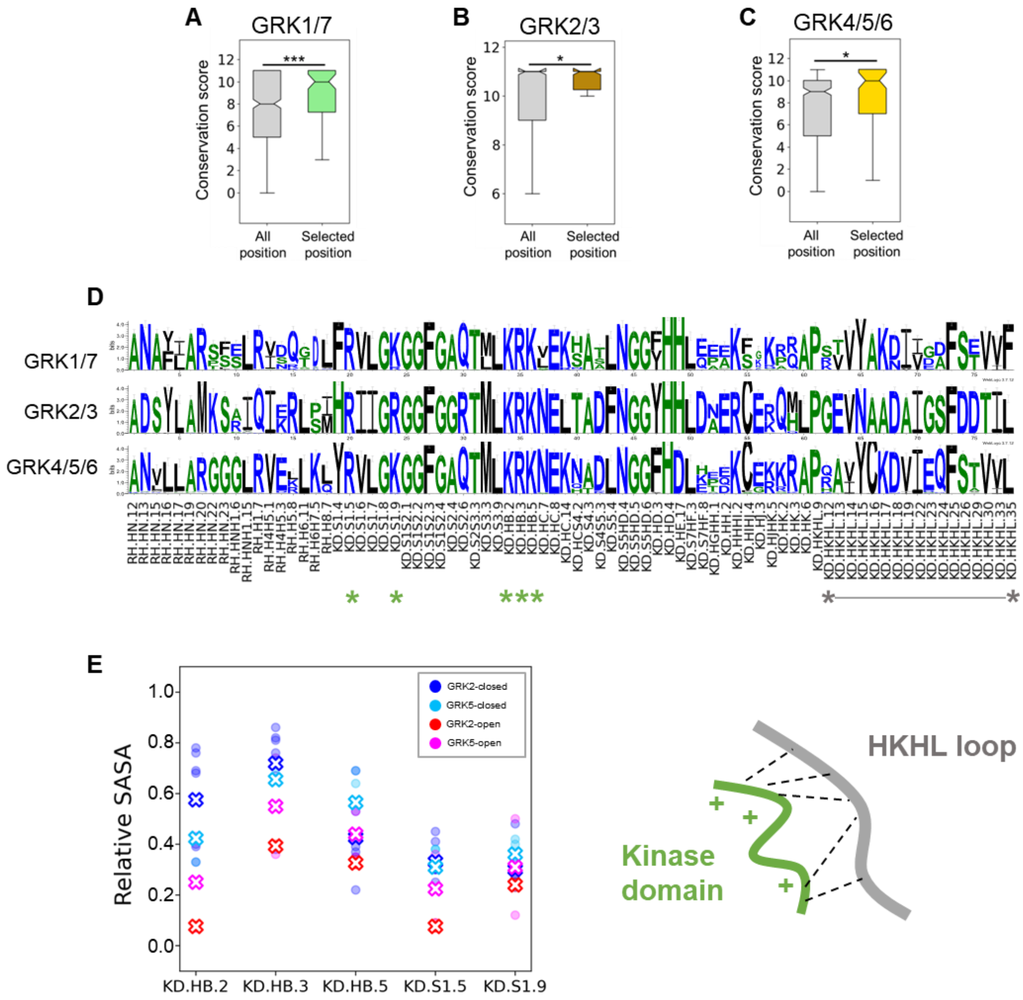

Supplementary figure 9. Conservation score comparison of all residues and the selected 78 positions (A)GRK1/7. (B)GRK2/3. (C)GRK4/5/6, Mann-Whitney U test was performed. (*: p-value < 0.1, **:p-value < 0.01, ***:p-value<0.001) (D) Sequence logo of common conformational movement GCL positions in Figure5A. asterisk mark present the positive charged amino acid and HKHL loop interaction residues. (E) Relative Solvent Accessible Surface Area (SASA) of each structure files. Blue: GRK2-closed, Red: GRK2-open, Cyan: GRK5-closed, Magenta: GRK2-open. ‘X’ marks are average point of each group.

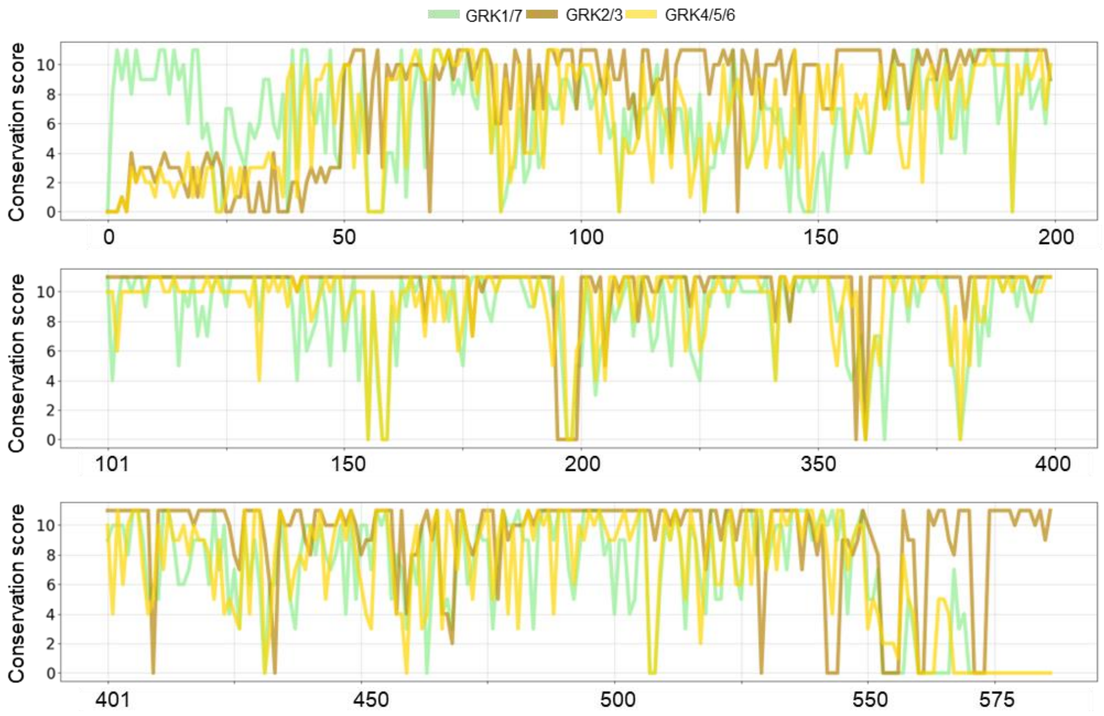

Supplementary figure 10. Conservation score of all GRK Common Label (GCL) positions according to subfamilies and overall GRKs. X-axis labels for GCL positions are in attached files.

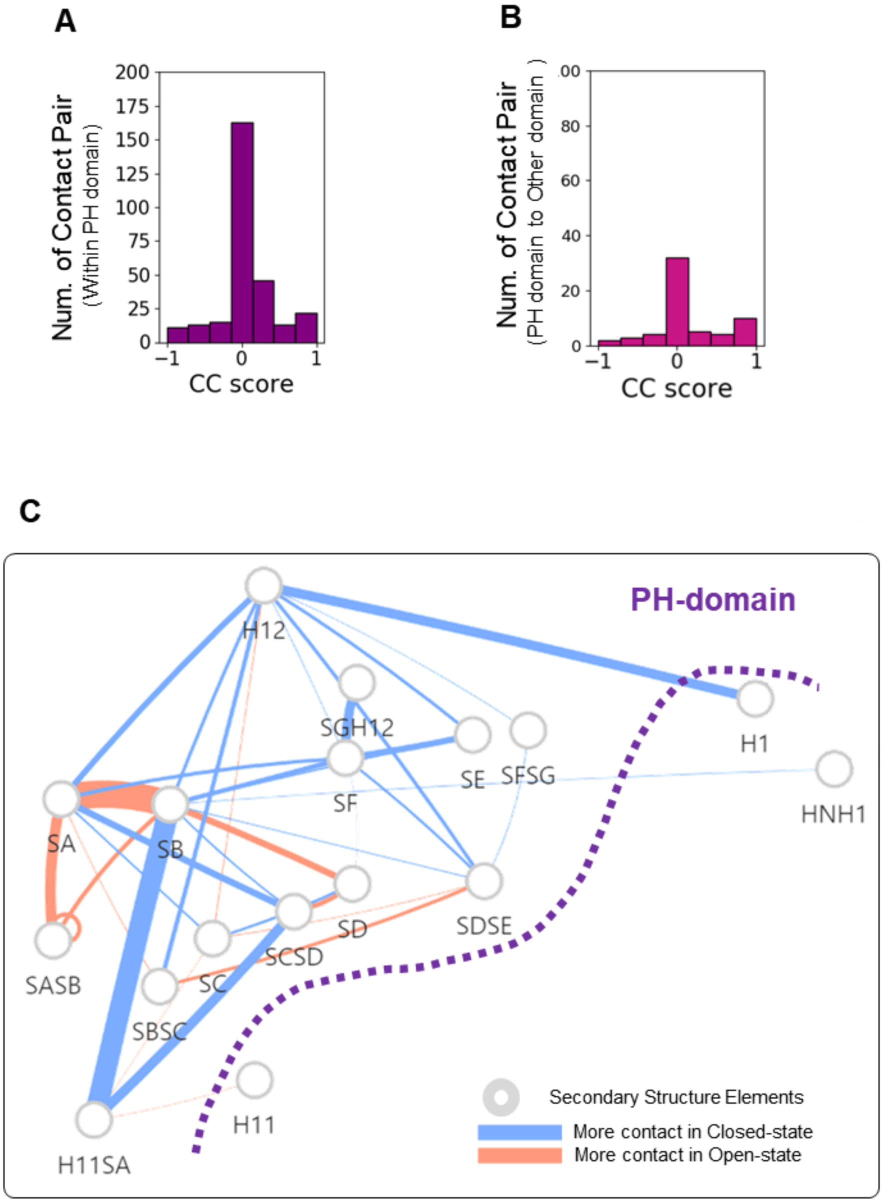

Supplementary figure 11. (A) Histogram of intra-PH domain conformational change score at residue level. (B) Histogram of PH domain to other domain Conformational Change score(CC score) at residue level (C) Network plot of conformational change involving PH domain. Nodes are secondary structure elements and edges showed strength of contacts.

Reference

1. Berglund, L. *et al.* A genecentric Human Protein Atlas for expression profiles based on antibodies. *Molecular & cellular proteomics* 7, 2019–2027 (2008).

2. Matthees, E. S. F., Haider, R. S., Hoffmann, C. & Drube, J. Differential regulation of GPCRs—are GRK expression levels the key? *Front Cell Dev Biol* 9, 687489 (2021).
